# Safety profile of trastuzumab deruxtecan in advanced breast cancer: Expert opinion on adverse event management

**DOI:** 10.1007/s12094-024-03383-x

**Published:** 2024-02-09

**Authors:** Eva Ciruelos, Jose Ángel García-Sáenz, Joaquín Gavilá, Miguel Martín, César A. Rodríguez, Álvaro Rodríguez-Lescure

**Affiliations:** 1 Medical Oncology Unit, Hospital 12 de Octubre, Madrid, Spain; 2 Medical Oncology Unit, Hospital Clínico San Carlos, Madrid, Spain; 3 Medical Oncology Unit, Fundación Instituto Valenciano de Oncología, Valencia, Spain; 4 Medical Oncology Unit, Hospital Gregorio Marañón, Madrid, Spain; 5 Medical Oncology Unit, Hospital Universitario de Salamanca-IBSAL, Salamanca, Spain; 6 Medical Oncology Unit, Hospital General Universitario de Elche, Alicante, Spain

**Keywords:** Adverse event management, Breast cancer, HER2, Trastuzumab deruxtecan

## Abstract

Trastuzumab deruxtecan (T-DXd) is an antibody–drug conjugate that targets human epidermal growth factor receptor 2 (HER2) and has shown promising results in the treatment of advanced/metastatic breast cancer. The objective of this report is to provide guidance on the prophylaxis, monitoring, and management of adverse events (AEs) in patients with breast cancer treated with T-DXd, and to emphasize that proper management of AEs is needed to optimize the effectiveness of T-DXd treatment and reduce the number of discontinuations. The article covers various aspects of T-DXd treatment, including its clinical efficacy, safety profile, and dosing considerations, and provides practical recommendations for managing AEs, such as nausea/vomiting, interstitial lung disease, and hematologic toxicity. Although there are still many knowledge gaps about the cause and incidence of AEs in real-world patients, this document may serve as a valuable resource for clinicians who are involved in the care of breast cancer patients receiving T-DXd treatment.

## Introduction

Trastuzumab deruxtecan (T-DXd; Enhertu®) is an antibody–drug conjugate targeting human epidermal growth factor receptor 2 (HER2). The molecule is composed of trastuzumab, a humanized anti-HER2 immunoglobulin G1 antibody, attached to deruxtecan (DXd), a potent topoisomerase I inhibitor, by a tetrapeptide-based cleavable linker [1, 2]. T-DXd has a high drug-to-antibody ratio and good stability, facilitating the internalization of the potent cytotoxic in HER2-positive cells upon reaching tumor tissue. Among other mechanisms, after lysosomal enzymes split the linker, the released DXd molecule causes DNA damage and apoptosis. In addition, T-DXd has high cell membrane permeability, which facilitates its dissemination in the tumour microenvironment and allows it to reach neighboring tumor cells regardless of the level of HER2 expression, an effect known as the bystander effect [3].

Currently, T-DXd is approved in Europe and the United States (US) for the treatment of adult patients with unresectable or metastatic HER2-positive breast cancer who have received at least one (Europe) or two (US) anti-HER2-based regimens [4, 5], based on the results from the DESTINY-Breast03, 02, and 01 trials. T-DXd has also been recently approved in Europe for the treatment of adult patients with unresectable or metastatic HER2-low breast cancer who have received prior chemotherapy in the metastatic setting or developed disease recurrence during or within 6 months of completing adjuvant chemotherapy [4], based on the results from the DESTINY-Breast04 trial. The initial treatment schedule for T-DXd is 5.4 mg/kg administered as an intravenous infusion once every 3 weeks (21-day cycle). Treatment is maintained until disease progression or unacceptable toxicity [4, 5].

T-DXd represents a highly efficacious treatment option for pretreated patients with HER2-positive and HER2-low advanced or metastatic breast cancer. Results from the phase III trials demonstrated a favorable benefit-risk profile. However, the emergence of T-DXd-related adverse events (AEs) may require temporary dose interruptions, dose reductions, or discontinuation of treatment according to the instructions described in the summary of product characteristics (SmPC) [4]. To maximize the efficacy of the drug, early or even prophylactic management of possible toxicities is desirable. In this review, we describe the clinical evidence for the efficacy and safety profile of T-DXd and provide a guide for the management of drug-related AEs in clinical practice, based on the authors’ experience. The objective is to provide guidance for the administration of proper prophylaxis and treatment, allowing patients to benefit from the proven efficacy of T-DXd.

## Efficacy results in phase III clinical trials

DESTINY-Breast02 was a randomized, open-label, phase III trial that evaluated the efficacy and safety of T-DXd versus treatment of physician’s choice (TPC; i.e., trastuzumab/capecitabine or lapatinib/capecitabine) in patients with HER2-positive unresectable and/or metastatic breast cancer previously treated with trastuzumab emtansine (T-DM1). A total of 608 patients were randomized 2:1 to receive either T-DXd or TPC [6]. The median (range) number of previous lines of systemic therapy in the metastatic setting was 2 (0–10) in the T-DXd arm and 2 (1–8) in the TPC arm. The median progression-free survival (PFS) was 17.8 months (95% confidence interval [CI] 14.3–20.8) for patients who received T-DXd (*n* = 406) compared with 6.9 months (95% CI 5.5–8.4) with TPC (*n* = 202; hazard ratio [HR] 0.3589; 95% CI 0.2840–0.4535; *P* < 0.0000001). Among the patients treated with T-DXd, 70% had an objective response, as compared with 29% of patients treated with TPC. Overall survival (OS) was also significantly longer for patients treated with T-DXd than in those who received TPC (39.2 vs. 26.5 months; HR 0.6578; 95% CI 0.5023–0.8605; *P* = 0.0021) [6].

The phase III DESTINY-Breast03 study compared the efficacy and safety of T-DXd versus T-DM1 in patients who had previously been treated with trastuzumab and a taxane for advanced/metastatic disease (*N* = 524) [7]. The median (range) number of previous therapy lines in the context of metastatic disease was 1 (0–16) in the T-DXd arm and 2 (0–14) in the T-DM1 arm. Median duration of study follow-up was 28.4 months with T-DXd and 26.5 months with T-DM1 [8]. The objective response rate was 79% in the T-DXd arm and 34% in the T-DM1 arm. Median PFS according to the independent central committee was 28.8 months (95% CI 22.4–37.9) with T-DXd compared with 6.8 months (95% CI 5.6–8.2) with T-DM1 (HR 0.334; *P* < 0.000) [8]. Median OS was not reached in the T-DXd or T-DM1 arms (95% CI 40.5 months to not estimable [NE] and 34.0 months to NE, respectively; HR 0.64; 95% CI 0.47–0.87; *P* = 0.0037) [8]. The OS rate at 12 and 24 months was also higher with T-DXd versus T-DM1 (94.1% vs. 86.0% and 77.4% vs. 69.9%, respectively) [8]. Subgroup analysis showed that the OS benefit was greater with T-DXd than with T-DM1 in all patient subgroups [8].

DESTINY-Breast04 was a randomized, open-label, phase III clinical trial that enrolled 557 patients with unresectable or metastatic HER2-low breast cancer [9]. The study included two cohorts of patients treated with at least one prior chemotherapy: 494 hormone receptor-positive (HR-positive) patients considered endocrine-refractory and 63 hormone receptor (HR)-negative patients. Patients were randomized (2:1) to receive either T-DXd or the physician’s chemotherapy choice (including eribulin, capecitabine, gemcitabine, nab-paclitaxel, or paclitaxel). Patients in both groups had received a median of three lines of treatment for metastatic disease. Median PFS in the HR-positive cohort (primary endpoint) was 10.1 months (95% CI 9.5–11.5) in the T-DXd arm and 5.4 months (95% CI 4.4–7.1) in the chemotherapy arm (HR 0.51; 95% CI 0.40–0.64; *P* < 0.0001). Median PFS in the overall population was 9.9 months (95% CI 9.0–11.3) in the T-DXd arm and 5.1 months (95% CI 4.2–6.8) for those receiving chemotherapy (HR 0.50; 95% CI 0.40–0.63; *P* < 0.0001). In the overall population, median OS was 23.4 months (95% CI 20.0–24.8) in the T-DXd arm versus 16.8 months (95% CI 14.5–20.0) in the chemotherapy arm (HR 0.64; 95% CI 0.49–0.84; *P* = 0.001). The percentage of patients with a confirmed objective response among all patients was 52.3% (95% CI 47.1–57.4) in the T-DXd arm and 16.3% (95% CI: 11.3 to 22.5) in the chemotherapy arm [9].

## Safety profile of T-DXd in phase III clinical trials

Table 1 and Table 2 show the main safety results from the DESTINY-Breast02, 03, and 04 trials. In the DESTINY-Breast 02 trial, the median duration of treatment was 11.3 months with T-DXd and 4.5 months with TPC. The incidence of serious AEs was 25.5% in the T-DXd arm and 23.6% in the TPC arm, and the incidence of grade ≥ 3 AEs was 53% and 44%, respectively [6]. The most common T-DXd-related AEs of any grade were nausea, vomiting, and alopecia, and the most common of grade ≥ 3 AEs were neutrophil count decreased, neutropenia, anemia, and nausea [6]. Drug-related interstitial lung disease (ILD)/pneumonitis occurred in 42 patients (10.4%) in the T-DXd arm, of which five patients (1.2%) had grade ≥ 3 events [10]. The patient-reported outcomes (PROs) from DESTINY-Breast02 suggested that the impact of T-DXd over time on nausea and vomiting was worse compared with TPC; however, the increase in nausea and vomiting scores with T-DXd was only clinically significant in early cycles [11].

**Table 1:** Overall safety summary of DESTINY-Breast03 and 04

Drug-related events, *n* (%)	DESTINY-Breast03		DESTINY-Breast04	
	T-DXd (*n* = 257)	T-DM1 (*n* = 261)	T-DXd (*n* = 371)	TPC (*n* = 172)
TEAEs	252 (98)	228 (87)	NA	NA
Serious TEAEs	33 (13)	20 (8)	NA	NA
TEAEs associated with drug discontinuations	51 (20)	17 (7)	60 (16)	14 (8)
TEAEs associated with dose interruptions	108 (42)	45 (17)	143 (38)	72 (42)
TEAEs associated with dose reductions	65 (25)	38 (15)	84 (23)	66 (38)
TEAEs associated with deaths	0	0	14 (4)	5 (3)

*NA* not available, *T-DM1* trastuzumab emtansine, *T-DXd* trastuzumab deruxtecan, *TEAE* treatment-emergent adverse event, *TPC* treatment of physician’s choice

**Table 2:** Treatment-emergent adverse events with T-DXd reported by ≥ 20% of patients in the DESTINY-Breast03, 04, and 02 studies

TEAE, *n* (%)	DESTINY-Breast03 *N* = 257		DESTINY-Breast04 *N* = 371		DESTINY-Breast02 *N* = 404	
	Any grade	Grade ≥ 3	Any grade	Grade ≥ 3	Any grade	Grade ≥ 3
Nausea	198 (77.0)	18 (7.0)	271 (73.0)	17 (4.6)	293 (72.5)	27 (6.7)
Vomiting	133 (51.8)	4 (1.6)	126 (34.0)	5 (1.3)	152 (37.6)	15 (3.7)
Neutropenia	79 (30.7)	41 (16.0)	123 (33.2)	51 (13.7)	144 (35.7)	74 (18.3)
Asthenia/fatigue	79 (30.7)	15 (5.8)	177 (47.7)	28 (7.5)	147 (36.4)	16 (4.0)
Alopecia	102 (39.7)	1 (0.4)	140 (37.7)	0	150 (37.1)	1 (0.2)
Anemia	95 (37.0)	24 (9.3)	123 (33.2)	30 (8.1)	115 (28.5)	32 (7.9)
Leukopenia	60 (23.3)	16 (6.2)	86 (23.2)	24 (6.5)	NA	NA
Anorexia (decreased appetite)	78 (30.4)	4 (1.6)	106 (28.6)	9 (2.4)	125 (30.9)	7 (1.7)
Thrombocytopenia	64 (24.9)	20 (7.8)	88 (23.7)	19 (5.1)	NA	NA
Diarrhea	83 (32.3)	3 (1.2)	83 (22.4)	4 (1.1)	109 (27.0)	11 (2.7)
Constipation	96 (37.4)	0	79 (21.3)	0	142 (35.1)	1 (0.2)
Of special interest						
ILD/Pneumonitis	39 (15.2)	2 (0.8)	37 (12.1)	8 (2.1)	42 (10.4)	5 (1.2)

*ILD* interstitial lung disease, *NA* not available, *TEAE* treatment-emergent adverse event

In the DESTINY-Breast03 trial, the median treatment duration was 18.2 months (interquartile range [IQR] 9.0–29.4) for T-DXd and 6.9 months (IQR 2.8–13.3) for T-DM1 [7]. AEs were common with T-DXd, and 56% of patients had grade ≥ 3 AEs. The most frequent AEs were neutropenia, anemia, nausea, thrombocytopenia, leukopenia, and fatigue. T-DXd was not associated with high rates of cardiotoxicity [7, 8]. The rate of treatment-emergent AEs (TEAEs) of any grade was similar in both arms (> 99% with T-DXd vs. 95% with T-DM1), as was the rate of grade ≥ 3 TEAEs (56% vs. 52%). However, exposure-adjusted rates were lower with T-DXd than with T-DM1 (grade ≥ 3 TEAEs: 0.36 vs. 0.65; serious TEAEs: 0.16 vs. 0.28). Drug-related ILD/pneumonitis was reported in 15.2% of patients receiving T-DXd and was the most common cause of treatment discontinuation in this arm; however, the number of high-grade ILD cases was low, with two cases (0.8%) of grade 3 ILD and none of grade 4/5 in the T-DXd arm [8]. PROs for the impact of nausea and vomiting showed that the time to definitive deterioration (TDD; defined as a ≥ 10-point change from baseline in the direction of deterioration for the specific score being used) was shorter for T-DXd than for T-DM1 (median TDD 7.3 months; 95% CI 4.4–12.5 vs. NE; 95% CI NE–NE; HR 2.11; 95% CI 1.6–2.8; *P* < 0.0001); for the first few treatment cycles, patients receiving T-DXd had higher levels of symptomatology in the nausea/vomiting subscale than those receiving T-DM1 [12].

In the DESTINY-Breast 04 trial, the median duration of treatment was 8.2 months (range 0.2 –33.3) and 3.5 months (range 0.3–17.6) in the T-DXd and TPC arms, respectively. The incidence of serious AEs was 27.8% in the T-DXd arm and 25.0% in the TPC arm, and the incidence of grade ≥ 3 AEs was 52.6% and 67.4%, respectively [8]. The most common T-DXd-related AEs of any grade were nausea, fatigue, and alopecia, and the most common of grade ≥ 3 AEs were neutropenia, anemia, and fatigue. Drug-related ILD/pneumonitis occurred in 45 patients (12.1%) who received T-DXd, of whom 13 (3.5%) had grade 1 events, 24 (6.5%) had grade 2, five (1.3%) had grade 3 and three (0.8%) had grade 5 [9].

## Experts’ recommendations for toxicity management

### Gastrointestinal toxicity

In general, nausea and vomiting are the most common side effects of anticancer therapies [13]. For T-DXd, as observed in phase III clinical trials, the incidence of any grade nausea was 72.5–77.0% and grade ≥ 3 nausea was 4.6–7.0%, while these values were 34.0–51.8% and 1.3–3.7%, respectively, for vomiting (Table 2). The median time to first onset was 2 days for nausea and 10 days for vomiting; the risk of the first event of nausea and vomiting was higher in earlier cycles, but the prevalence was relatively consistent over time [14]. The experience of patients treated in Spain suggests that, indeed, the emetogenic effect of T-DXd is frequent, but different from the usual acute emesis profile of topoisomerase I inhibitors, such as irinotecan. The emesis is usually of grade 1/2 but is sustained for many days during the cycle, which may affect the quality of life of patients due to its persistence. This was an unexpected pattern, which needs to be investigated in future studies in terms of pathophysiology, duration, and antiemetics effects between cycles.

**Fig. 1:**
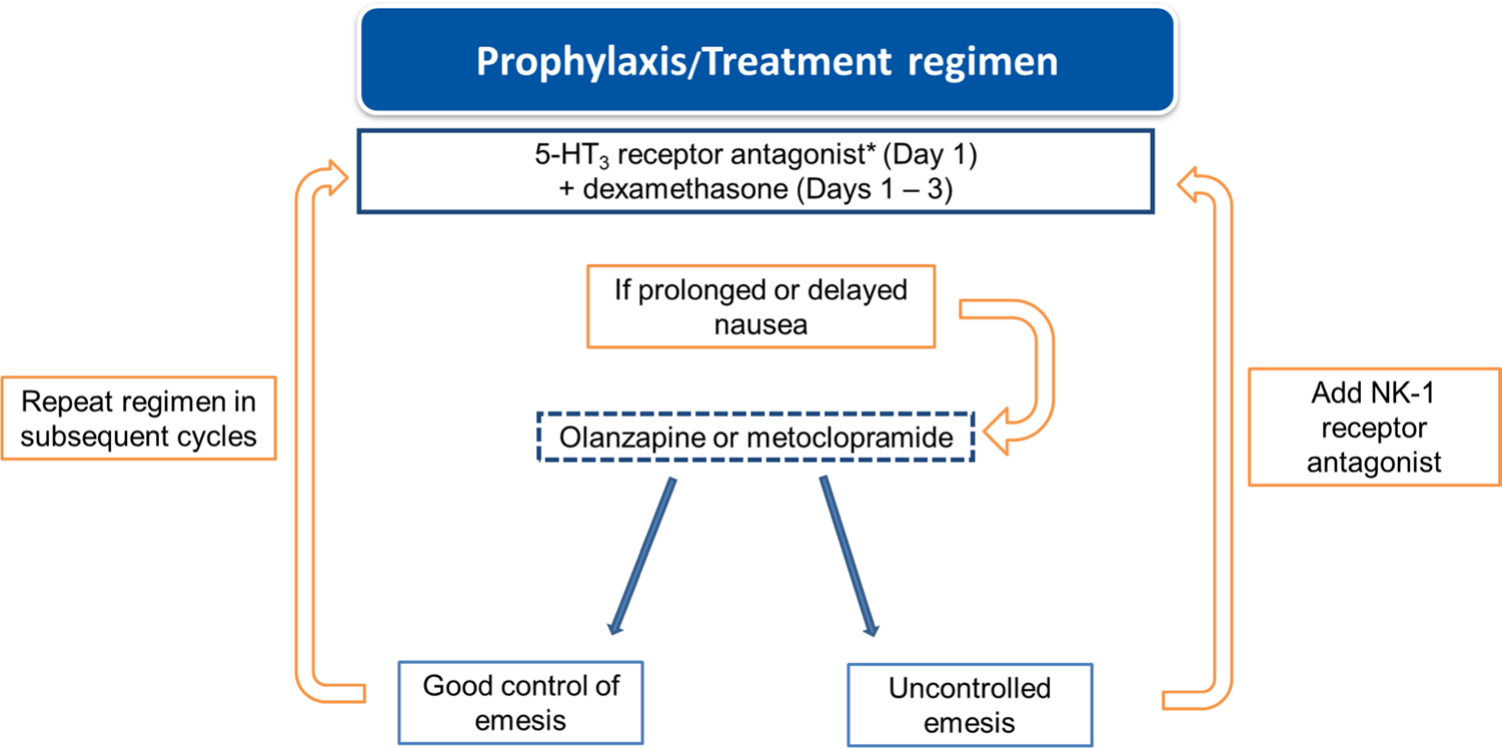
Management of T-DXd-related emesis. *Preferred option: palonosetron. *5-HT3* serotonin type 3, *NK-1* neurokinin-1

Although a different pattern from the classical emetic effect, we consider T-DXd emesis to be of moderate risk. This is consistent with some clinical guidelines [15, 16], although others consider T-DXd as highly emetogenic [17]. Apart from modifying dietary habits, we recommend administering antiemetic prophylaxis with the combination of a 5-HT_3_ receptor antagonist (preferably palonosetron, if available) on day 1 and a corticosteroid (dexamethasone) on days 1–3. However, this management strategy is based on a moderate risk of emesis, and may fall short in some cases [18]; in case of persistent symptoms on days 1–5, the addition of a neurokinin-1 receptor antagonist (aprepitant) could be considered for uncontrolled emesis (Fig. 1). Since protracted nausea has been described by many investigators beyond day 5, olanzapine 5–10 mg at nighttime can also be used. If nausea/vomiting reaches grade 3 despite antiemetic therapy, the next T-DXd infusion may be delayed until grade 1 nausea/vomiting is achieved, maintaining the dose if resolution occurs within 1 week, and reducing the dose by one level if resolution is later [19]. In patients with anticipatory emesis, the use of benzodiazepines prior to treatment should be considered.

Based on the phase III clinical trials, grade 3 diarrhea was a rare toxicity, although almost one-third of patients suffered low-grade diarrhea. We advise modification of lifestyle and dietary habits (preferencing low-fat meals) and prescription of loperamide if grade 2 diarrhea occurs. For cholinergic (acute) diarrhea, atropine should be administered. Regarding constipation, there were no episodes of grade ≥ 3 events in the clinical trials (Table 2). In our opinion, grade 1 or 2 constipation is usually associated with the use of 5-HT_3_ antagonists for nausea.

### Lung toxicity

ILD describes a heterogeneous series of pulmonary parenchyma disorders manifested as inflammation and fibrosis of the pulmonary interstitium [14]. ILD radiographic patterns include nonspecific interstitial pneumonia, usual interstitial pneumonia, acute interstitial pneumonia, and organized pneumonia [20]. Radiological diagnosis is essential for adequate management. The imaging technique of choice is chest computed tomography (CT), particularly high-resolution CT, due to its high sensitivity and specificity, and its ability to assess the extent of lung involvement. Up to one-third of patients with ILD may be asymptomatic, so an incidental diagnosis may occur in patients with radiological evidence of interstitial pneumonia [21].

The T-DXd SmPC warns of the occurrence of ILD/pneumonitis cases, some of them with a fatal outcome, in phase I and II clinical trials [2, 22], and provides T-DXd dose modification guidelines [4]. In the phase III trials, the majority of cases (103 out of 118 reported) were grade 1 or 2 due to the implementation of early detection and treatment measures. It is important to know the expected frequency of this AE, which seems to be 10–15%, while the median time to occurrence of the event was 29.9 weeks (IQR 12.3–48.0) in DESTINY-Breast02 [6], 8.1 months (IQR 4.2 –15.0) in DESTINY-Breast03 [8], and 129 days (range 26–710) in DESTINY-Breast04 [9]. The incidence was similar (10.9%) in a T-DXd safety meta-analysis including 1457 patients with different types of tumors; the median time to the onset of ILD and pneumonitis was 43 days (range 1–350) and 55 days (range 35–133), respectively [23]. A pooled safety analysis of nine studies in patients with various cancers (*N* = 1150) showed that most episodes of T-DXd-related ILD were low-grade (77% were grade 1 or 2) and occurred within the first 12 months of treatment [24]. The risk factors associated with ILD development included age (> 65 years), low baseline oxygen saturation (< 95%), time since diagnosis (> 4 years), lung comorbidities (asthma, chronic obstructive pulmonary disease, prior ILD/pneumonitis, pulmonary fibrosis, pulmonary emphysema, and radiation pneumonitis), and renal insufficiency [24].

**Fig. 2:**
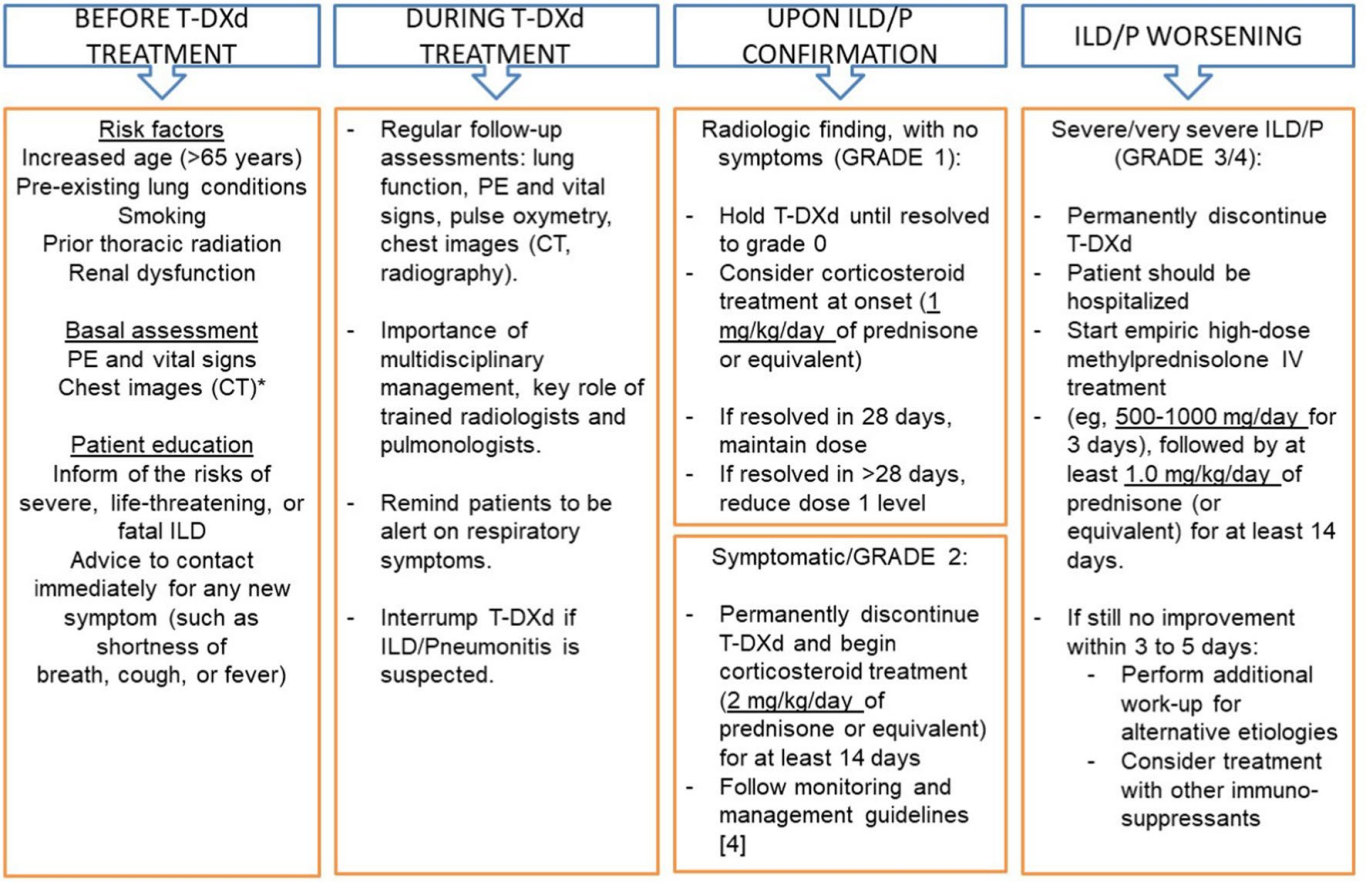
Essential elements of proactive monitoring for interstitial lung disease/pneumonitis early detection and management. *Ideally, high-resolution CT. *CT c*omputed tomography, *ILD/P* interstitial lung disease/pneumonitis, *IV* intravenous, *PE* physical examination

Evidence with other drugs suggests that a longer time between the onset of ILD/pneumonitis and drug discontinuation may be associated with worse outcomes [25]. Conversely, early identification as grade 1 or asymptomatic (Table 3) will allow for the administration of timely and effective treatment, and other published guidelines recommend multidisciplinary, proactive management and ILD-related education in both patients receiving T-DXd and their healthcare providers [26]. Aligned with this, we also place an emphasis on proactive monitoring of each patient and recommend the following (Fig. 2): (1) a thorough assessment of individual risk factors (history, respiratory comorbidities); (2) initial and regular evaluation of lung function, in addition to vital signs, physical examination, and chest imaging (preferably, baseline and follow-up high-resolution CTs, with intervals of 9–12 weeks); (3) the implementation of a diagnostic and therapeutic algorithm, with multidisciplinary collaboration and trained radiologists; and (4) adequate information to patients about the risks of ILD/pneumonitis and its clinical manifestations, so that they will visit their doctor promptly if new onset or worsening of symptoms occur, such as dyspnea, fever, or cough [18, 27]. In addition, the oncology nurse should check for respiratory symptoms at each visit and/or oxygen levels measured by pulse oximetry. Physicians must stay alert throughout treatment, as the timing for the presentation of pulmonary toxicity is highly variable, which makes it very difficult to diagnose. Onset can happen within weeks to months of initiating T-DXd, and it can present with the first cycle or any subsequent treatment courses, mainly during the first year of therapy. Thus, close monitoring may facilitate early recognition of ILD/pneumonitis (grade 1), which is important so that T-DXd therapy can be discontinued, corticosteroids initiated (see Fig. 2 for recommended doses), and higher-grade ILD/pneumonitis prevented. In case of confirmation of grade 2 ILD/pneumonitis, it is mandatory to refer the patient to a pulmonologist consultation, perform differential diagnosis (e.g., opportunistic co-infections that would require antibiotic therapy), bronchoscopy, and bronchial lavage. If there is no response to corticosteroids, empirical use of other drugs, such as mycophenolate or infliximab, could be considered.

**Table 3:** Drug-induced ILD/pneumonitis grades according to CTCAE version 5.0

Grade	Clinical severity
Grade 1 (mild)	Asymptomatic patient with radiographic findings only, not present at baseline
Grade 2 (moderate)	Mild respiratory symptoms that do not deteriorate the patient’s quality of life
Grade 3 (severe)	Symptoms that lead to a worsening of the quality of life and limit the activities of daily living of the patient, possibly needing oxygen therapy, regardless of the severity of the radiologic findings
Grade 4 (very severe, life-threatening or disabling)	Severe, disabling symptoms leading to patient’s hospitalization and requirement for mechanical ventilatory support
Grade 5 (fatal)	Death

*CTCAE* Common Terminology Criteria for Adverse Events

Evidence for treatment with T-DXd is lacking for certain profiles of patients with previous lung conditions, as they were excluded from clinical trials of T-DXd. Similarly, patients with a medical history of (non-infectious) ILD/pneumonitis that required steroids or with active or suspected ILD/pneumonitis were excluded from the studies. The benefit-risk of T-DXd treatment in this patient population should be carefully assessed, and management of lung toxicity on treatment should be individualized. In patients at higher risk of developing ILD or with considerable lung involvement, steroids are recommended to manage grade 1 ILD. In addition to this, if grade 1 ILD does not improve upon initiation of corticosteroids, guidelines for managing grade 2 ILD should be followed [19]. Patients with a history of ILD/pneumonitis or patients with moderate or severe renal impairment may be at increased risk of developing ILD/pneumonitis and should be monitored carefully [4]. In patients receiving moderate doses (prednisone > 20 mg/day or equivalent) of steroids (mainly patients with brain metastases), adequate *Pneumocystis jirovecii* prevention must be undertaken with a prophylactic antibiotic (e.g., trimethoprim-sulfamethoxazole). This could help to reduce the risk of infection by this pathogen, which is the first option for differential diagnosis of ILD [28].

### Asthenia/fatigue

Oncology patients often experience fatigue during cancer treatment, which negatively affects their quality of life. This fatigue is a complex phenomenon that differs from occasional tiredness in that it is not relieved by rest or sleep [29]. In the DESTINY-Breast trials, the incidences of asthenia or fatigue (any grade) were 30.7–47.7%, while grade ≥ 3 events were reported in 4.0–7.5% of patients (Table 2). The median time to first onset of fatigue was 22 days, and its incidence was relatively consistent across cycles [14]. No grade 4 or 5 fatigue events were reported in DESTINY-Breast03 [8].

This AE is very prevalent in the metastatic scenario; it is associated with chemotherapy and can have several underlying causes. We highlight as possible etiologies the presence of anemia, hyporexia, persistent nausea, hypothyroidism, and even insomnia. Beyond these potentially treatable causes, which need to be addressed, the management of asthenia is complex and heterogeneous. In line with the available scientific evidence [30, 31], we suggest that asthenia can be controlled through the implementation of non-pharmacologic measures. As per other guidelines, we consider the implementation of educational measures, guided therapeutic physical exercise and energy conservation techniques to be paramount, provided that they are carried out within the framework of a functional rehabilitation program [32]. The impact of dose reductions on asthenia improvement has not been studied, but we recommend dose reduction when grade 2 asthenia does not improve with rest and is negatively affecting quality of life.

### Hematologic toxicity

T-DXd-related cytopenia, similar to that occurring with conventional chemotherapy, suggests an off-target effect of the cytotoxic agent [33]. The median time to first occurrence of hematologic toxicity in the DESTINY-Breast03 study was 64 days for neutropenia, 70 days for anemia, and 132 days for thrombocytopenia. The overall rates of hematologic TEAEs decreased over time and were highest in the first few cycles [14]. In terms of frequency, neutropenia was the most common (30.7–35.7% of any grade and 13.7–18.3% of grade ≥ 3; only 1% were of grade 4 severity); febrile neutropenia occurred in 1.4% of patients and was categorized as a common adverse effect according to the SmPC (≥ 1/100 to < 1/10) [4]. However, recent analyses have shown a lower incidence of febrile neutropenia [14]. We suggest delaying the next T-DXd infusion for up to 2 weeks in cases of grade 3 neutropenia and reducing the dose if neutropenia recurs. If this strategy does not improve neutropenia, a colony-stimulating factor may be given to patients with persistent or complicated grade 3 neutropenia [19]. In cases of grade 4 neutropenia, we recommend first reducing the dose if the neutropenia is not complicated, since this is a good option to maintain the patient’s quality of life. If neutropenia does not resolve with dose reduction and neutrophils are between 1,000 and 1,500/mm^3^, then we recommend administering colony-stimulating factors, always based on clinical judgement. In the event of febrile neutropenia, the first step should be to interrupt T-DXd treatment and initiate antibiotic therapy; subsequent infusions should be administered at a reduced dose.

Approximately one-third of the patients in the phase III trials experienced anemia of any grade (Table 2), mainly in the first few cycles, which means that many of them may have had hemoglobin levels close to 8.1 g/dL and were symptomatic (provoking fatigue or asthenia). There were no grade 4 anemia events reported in DESTINY-Breast 03 [8]. It should be noted that treatment with T-DXd can be long-term, and that low-grade but symptomatic anemia hampers the quality of life of patients and their tolerance to treatment. We believe that this type of anemia does not usually respond to iron supplementation because patients are generally not iron-deficient and may require T-DXd dose reductions when they lead to asthenia. Data on the rate of blood transfusions in phase III clinical trials are lacking, but we recommend erythrocyte transfusions when anemia provokes asthenia or hemoglobin falls to < 10 g/dL, or erythropoietin administration as a last resort, according to clinical judgement.

As for thrombocytopenia, it is a condition that can persist after treatment with T-DM1 as a previous line and was reported more frequently at the first cycle (10.9%) than at the second (2.7%) and third (1.6%) cycles in the DESTINY-Breast 03 trial [14]. Grade 4 thrombocytopenia was rare (< 1%) [8]. Prior to each dose and as clinically indicated, routine complete blood counts should be conducted for the early detection of potential haematological adverse reactions. In the case of recurrent grade 3 thrombocytopenia, dose reductions should be considered.

### Other AEs of interest

DXd is a cytotoxic chemotherapeutic agent that affects proliferating tumor cells and other normally proliferating cells, such as those in the hair matrix (in the anagen phase, 90% of the time) [34]. In the DESTINY-Breast03 and 04 trials, around 37% of patients experienced some degree of alopecia, so it is important to inform patients of the possibility of hair loss during treatment. This AE first occurred after a median of 27 days of treatment and its prevalence was relatively consistent over time [14]. Although scalp cooling during infusion of a cytotoxic drug is an option for some chemotherapy schedules to minimize the alopecic effect, this strategy may not be effective with T-DXd because of the continuous release of the drug. There is an ongoing clinical trial comparing the rates of hair loss in people with metastatic breast cancer who use scalp cooling versus those who do not use scalp cooling after receiving standard-of-care treatment with T-DXd (NCT04986579). Until the results are available, we suggest considering a therapy such as minoxidil (0.5–2.5 mg/day) to reduce hair loss, although supporting evidence is lacking and prospective studies are needed.

Regarding trastuzumab-related cardiotoxicity, which is due to the expression of the HER2 receptor in cardiomyocytes, this AE usually manifests as an asymptomatic reduction in left ventricular ejection fraction (LVEF) [35]. The cases in the DESTINY-Breast03 trial (2.3%) were all asymptomatic and most resolved without intervention [7]. In DESTINY-Breast04, the frequency of left ventricular dysfunction was higher (all grades: 4.3%; grade 3: 0.3%) [9]. The SmPC includes a warning for left ventricular dysfunction [4], suggesting conventional cardiac function testing to assess LVEF prior to initiating T-DXd therapy and at regular intervals (3–4 months) during treatment, as clinically indicated. We recommend more exhaustive monitoring in patients with elevated cardiac risk (e.g., with a history of other drug-related cardiac events). Table 4 describes our recommendations regarding continuation, interruption, or discontinuation of T-DXd therapy according to the degree or severity of LVEF changes. A cardiologist should be consulted in the case of reduced LVEF [19], and the diagnostic technique should always be the same for all assessments. We consider that treatment discontinuation may not be necessary for asymptomatic patients, even if there is an absolute decrease of > 20% from baseline, because some patients have an initially high LVEF. In these specific cases, and in agreement with the cardiologist, treatment could be restarted if the patient recovers, and the expected benefit outweighs the risk. If the patient is monitored with a multi-gated acquisition (MUGA) scan and shows a clinically significant reduction of < 40% or > 20% MUGA difference, echocardiography and a complete cardiac evaluation may be recommended. Monitoring of serum markers is useful in patients undergoing prolonged treatments, as in the case of T-DXd. Thus, the assessment of predictive markers of chronic cardiac injury such as brain natriuretic peptide (BNP) and *N*-terminal pro-BNP could be considered [36].

**Table 4:** Summary of product characteristics guidelines for left ventricular ejection fraction management

Adverse event	Treatment modification
LVEF > 45% and absolute decrease from baseline of 10–20%	• Continue treatment with T-DXd
LVEF 40–45% and absolute decrease from baseline of < 10%	• Continue treatment with T-DXd • Repeat LVEF assessment within 3 weeks
LVEF 40–45% and absolute decrease from baseline of 10–20%	• Hold T-DXd treatment • Repeat LVEF assessment within 3 weeks • If LVEF has not recovered to within 10% of baseline, permanently discontinue T-DXd • If LVEF recovers to within 10% of baseline, resume treatment with T-DXd at the same dose
LVEF < 40% and absolute decrease from baseline of > 20%	• Hold T-DXd treatment • Repeat LVEF assessment within 3 weeks • If LVEF < 40% is confirmed or the absolute decrease from baseline is > 20%, permanently discontinue T-DXd
Symptomatic CHF	• Permanently discontinue T-DXd

*CHF* congestive heart failure, *LVEF* left ventricular ejection fraction, *T-DXd* trastuzumab deruxtecan

Another AE frequently reported in DESTINY-Breast03 and 04 was anorexia or decreased appetite (in 26.1–28.6% of patients). This condition may increase mortality, reduce treatment effects, and cause severe psychological distress in patients and their families [37]. It can also act as an underlying cause of fatigue. Although there are no specific measures to target T-DXd-related anorexia, the most effective way to decrease the incidence of this AE is to prevent nausea and vomiting, in addition to following the general recommendations for anorexia treatment, including renutrition, nutritional counselling, and cognitive-behavioral therapy [38]. However, there is no evidence to support the efficacy and safety of orexigenic drugs to manage T-DXd-related anorexia. Patients should be advised to maintain an adequate and nutritional food intake, to optimize health status and improve their prognosis. An individualized diet plan could be of help to achieve this [39, 40].

## Future perspectives and conclusions

There are still many knowledge gaps about the causes and incidence of T-DXd-related AEs in real-world patients, and the optimal management of many of these AEs. We also do not know the attributable role of the DXd payload or the trastuzumab antibody in the development of toxicity. The use of T-DXd will continue to increase in the near future, and knowing what AEs to expect and how to manage them effectively will become even more important. In the particular case of nausea/vomiting, experts consider that the temporal pattern of onset and persistence is unexpected and that it would be appropriate to describe the course of emesis (rather than its severity, which is already reported in the clinical trials) to implement an evidence-based approach. This would require a study collecting information from the patient’s point of view (PROs), with a daily questionnaire on quality of life and adverse effects. Another aspect that needs to be clarified is the efficacy of T-DXd in those patients who received reduced or interrupted dosing if this had an impact on the oncologic outcomes. In addition, the possible link of some biomarkers or polymorphisms with the occurrence of adverse effects, such as ILD/pneumonitis, should be investigated.

In our opinion, there is a high inter-patient variability in the incidence and severity of AEs, while this does not seem to be the case for antitumor activity, which is generalized. It is important that patients are aware of the potential toxicities associated with T-DXd, but also of its proven efficacy, so that their motivation to continue treatment is greater than the discomfort caused by adverse effects. Close monitoring and appropriate support can help prevent treatment discontinuation and maximize the benefits of T-DXd.

## Data Availability

Not applicable. This review article does not contain any unpublished data.
